# High Prevalence of Bovine Tuberculosis in Dairy Cattle in Central Ethiopia: Implications for the Dairy Industry and Public Health

**DOI:** 10.1371/journal.pone.0052851

**Published:** 2012-12-28

**Authors:** Rebuma Firdessa, Rea Tschopp, Alehegne Wubete, Melaku Sombo, Elena Hailu, Girume Erenso, Teklu Kiros, Lawrence Yamuah, Martin Vordermeier, R. Glyn Hewinson, Douglas Young, Stephen V. Gordon, Mesfin Sahile, Abraham Aseffa, Stefan Berg

**Affiliations:** 1 Armauer Hansen Research Institute, Addis Ababa, Ethiopia; 2 National Animal Health Diagnostic and Investigation Center, Sebeta, Addis Ababa, Ethiopia; 3 School of Veterinary Medicine, University College Dublin, Dublin, Republic of Ireland; 4 Centre for Molecular Microbiology and Infection, Imperial College London, London, United Kingdom; 5 Department for Bovine Tuberculosis, Animal Health and Veterinary Laboratories Agency, Weybridge, Surrey, United Kingdom; 6 Swiss Tropical and Public Health, Basel, Switzerland; Institut national de la santé et de la recherche médicale - Institut Cochin, France

## Abstract

**Background:**

Ethiopia has the largest cattle population in Africa. The vast majority of the national herd is of indigenous zebu cattle maintained in rural areas under extensive husbandry systems. However, in response to the increasing demand for milk products and the Ethiopian government's efforts to improve productivity in the livestock sector, recent years have seen increased intensive husbandry settings holding exotic and cross breeds. This drive for increased productivity is however threatened by animal diseases that thrive under intensive settings, such as bovine tuberculosis (BTB), a disease that is already endemic in Ethiopia.

**Methodology/Principal Findings:**

An extensive study was conducted to: estimate the prevalence of BTB in intensive dairy farms in central Ethiopia; identify associated risk factors; and characterize circulating strains of the causative agent, *Mycobacterium bovis*. The comparative intradermal tuberculin test (CIDT), questionnaire survey, post-mortem examination, bacteriology, and molecular typing were used to get a better understanding of the BTB prevalence among dairy farms in the study area. Based on the CIDT, our findings showed that around 30% of 2956 tested dairy cattle from 88 herds were positive for BTB while the herd prevalence was over 50%. Post-mortem examination revealed gross tuberculous lesions in 34/36 CIDT positive cattle and acid-fast bacilli were recovered from 31 animals. Molecular typing identified all isolates as *M. bovis* and further characterization by spoligotyping and MIRU-VNTR typing indicated low strain diversity within the study area.

**Conclusions/Significance:**

This study showed an overall BTB herd prevalence of 50% in intensive dairy farms in Addis Ababa and surroundings, signalling an urgent need for intervention to control the disease and prevent zoonotic transmission of *M. bovis* to human populations consuming dairy products coming from these farms. It is suggested that government and policy makers should work together with stakeholders to design methods for the control of BTB in intensive farms in Ethiopia.

## Introduction

The population of Ethiopia has increased dramatically in the last two decades, growing from approximately 55 million people in 1992 to a current estimate of around 85 million [1]. Increased population size has led to an inexorable increase in demand for food, putting pressure on the agricultural sector in which 85% of the work force is employed. Ethiopia has the largest livestock population in Africa, including an estimated ∼52 million cattle [2] that contributes to the livelihoods of 60–70% of the population [3]. The vast majority of the cattle are indigenous zebu (*Bos indicus*) managed under traditional husbandry systems (grazing in the field) in rural areas. However, in recent years the number of dairy cattle of highly productive exotic (*Bos taurus*, mainly Holstein-Friesian) and cross breeds has been on the rise, particularly in urban and peri-urban areas in response to the increasing demand for milk products and the Ethiopian government's effort to improve productivity in the livestock sector. The population of dairy cows accounts for 6.3 million animals (around 12% of the total cattle population) and the estimated total national milk production per year is 2.6 billion litres [4] of which the urban and peri-urban dairy farmers produce 2%.

In a country such as Ethiopia, where livestock are extremely important for people's livelihood, animal diseases can be a real threat to animal productivity and thus negatively impact on the agricultural sector and economic development. Bovine tuberculosis (BTB), caused by *Mycobacterium bovis*, is a chronic and contagious disease of cattle and other domestic and wild animals [5], [6]. BTB is prevalent worldwide but prevalence data is scarce in most developing countries due to lack of active control programmes. Several studies conducted since 2006 have confirmed that BTB is endemic in Ethiopia with prevalence rates varying from 0.8% to around 10% in extensive rural farming systems [7]–[11], while higher prevalence rates have been reported from regions in Ethiopia where intensive husbandry systems are more common [12]–[14]. As well as causing a high morbidity, BTB can also be a financial burden to farmers owning infected cattle; it has been suggested that cattle with BTB have a reduced productivity affecting milk yield and carcass value [15] as well as through reduced pulling power in traditional farming system [16]. BTB has also zoonotic potential [17], [18] - mainly through consumption of unpasteurised milk products - and its prevalence in Ethiopian cattle can therefore be a contributing factor to the human burden of TB in Ethiopia that currently is ranked as the 7^th^ highest in the world [19], [20].

This study was designed to get a better understanding of the prevalence of BTB among intensive dairy farms in the urban and peri-urban belt of Addis Ababa (central Ethiopia), areas from where the vast majority of commercial dairy products in the country are produced. Furthermore, we show molecular typing results of *M. bovis* strains circulating in this region of Ethiopia and discuss the importance of our findings for informing BTB control policies in Ethiopia.

## Materials and Methods

### Study sites and ethical considerations

**Figure 1:**
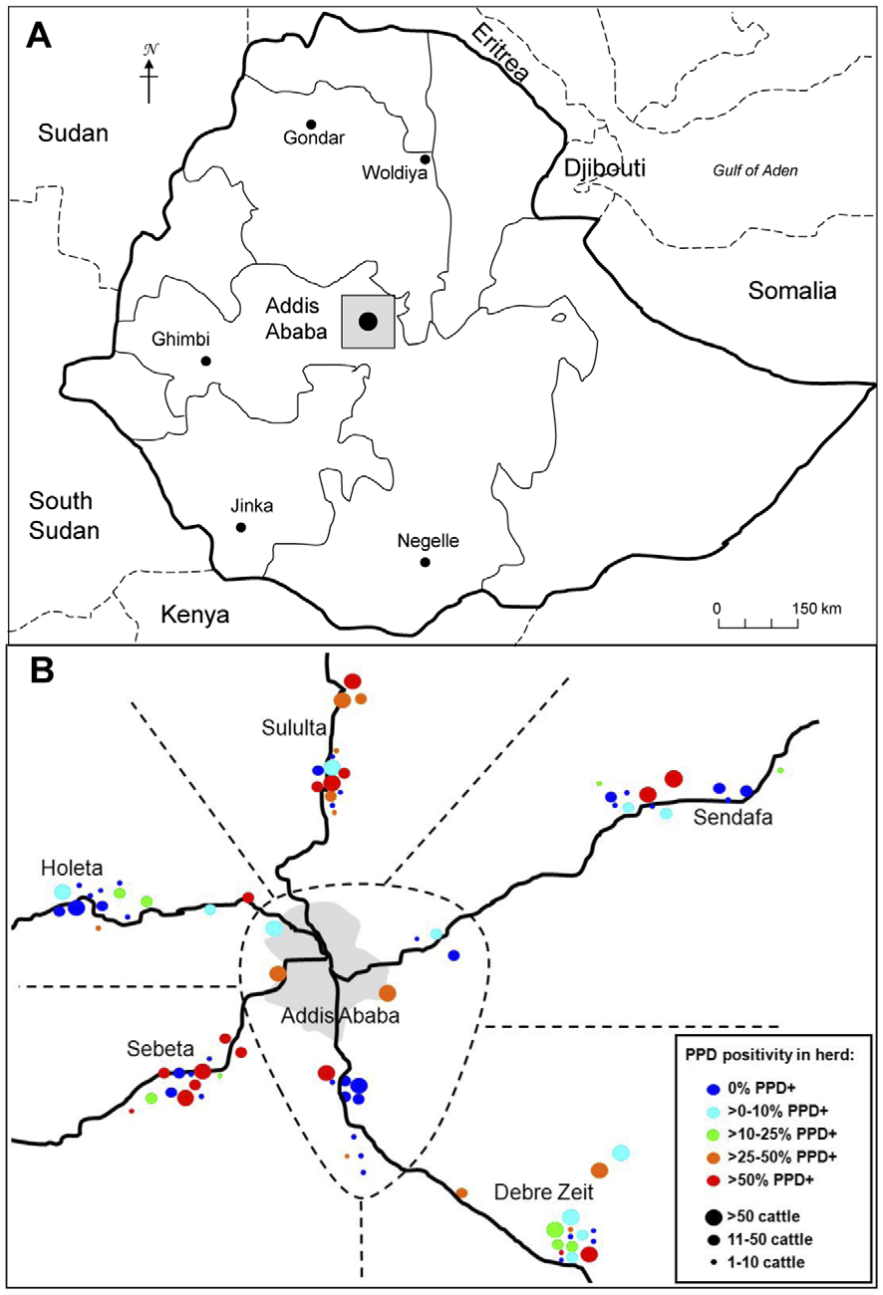
Mapping of (A) the study area (marked in grey) and other sites in Ethiopia where *M. bovis* isolates used in this study were collected, and (B) BTB prevalence in investigated farms in the five study areas. PPD positivity and farm size category are defined by colour and size of respective circle.

Statistics from the Ethiopian Ministry of Agriculture and Rural Development (MoARD) as well as data from regional agricultural offices recognised a high density of intensive dairy farms in a 50 km radius region from Addis Ababa city centre. Most dairy farms in this region had access to one of the five main roads that lead into the capital. Therefore, the region was divided into six geographically defined study areas, including Addis Ababa city, Debre Zeit, Sebeta, Holeta, Sululta and Sendafa (Figure 1). The recruitment of dairy farms within each study area was based on the existence of high numbers of Holstein-Friesian and cross breed cattle, as well as variation in herd size, with the latter being categorized into three groups; small herds (1–10 cattle), medium herds (11–50 cattle), and large herds (>50 cattle). This study was approved by the MoARD and was based on voluntary participation, and consent to participate was given from all farm owners after being informed about the study by local agricultural office authorities as well as by study representatives. Ethical approval for the study was given by the institutional ethical committee at the Armauer Hansen Research Institute (AHRI) and the All Africa Leprosy, Tuberculosis and Rehabilitation Training centre (ALERT). As part of the commitment of this study, one week training was given to 63 of the farm owners and/or their staff by professionals from National Animal Health Diagnostic and Investigation Center (NAHDIC), AHRI, the National Artificial Insemination Center (NAIC), and the Ministry of MoARD Extension Directorate and Oromia Special Zone Surrounding Finfinne Livestock Development & Marketing Agency Office. The training was done in the Amharic language and included topics such as mastitis, abortion, dystocia, calcium deficiency, BTB, brucellosis, heat detection, artificial insemination, vaccination, and herd management (e.g. annual planning, dairy management).

### Comparative intradermal test and questionnaire

All cattle older than six months within a herd, except clinically sick animals and cows one month pre-and post partum, were tested by CIDT: Two sites, 12 cm apart, horizontally of the mid-neck of the animal were shaved and the skin thickness was measured with a calliper. In calves with short neck, the sites were shaven vertically. Aliquots of 0.1 ml of 2500 IU/ml bovine purified protein derivative (PPD) and 0.1 ml of 2500 IU/ml avian PPD (Synbiotics Corporation, Lyon, France) were injected separately into the respective shaved site. The thickness of the skin at each injection site was measured again after 72 hours. The test results were interpreted in line with OIE recommendations (2004): if the difference between skin thickness at the bovine site of injection and the avian site of injection was <2 mm, between 2 mm and ≤4 mm, or >4 mm, the animal was classified as negative, doubtful (inconclusive), or positive for BTB, respectively. A herd with at least one positive reactor was considered as “PPD positive”. *M. avium* positivity was defined as previously described [9] with the avian PPD injection site showing a reaction of >4 mm between the testing day and the reading day. In addition, we calculated how many animals showed an avian skin reaction of >4 mm as compared to a bovine skin reaction [(A2-A1) - (B2-B1)>4 mm]. Information on farm structure and management were collected from 80 out of the 88 investigated farms using a standardised questionnaire (Questionnaire S1) with closed questions. The owner or manager of each farm was interviewed in the local language at the time the CIDT was performed. Sanitation status was judged as poor, medium (satisfactory), or good, based on aspects such as odour, waste drainage, cleanness of floor and animals, barn ventilation and light source, and animal stocking.

### Ante and post mortem examination

All cattle tested by CIDT were recorded for age, sex, breed, and given a body condition score (poor, medium, or good). Selected CIDT positive cattle from farms of all six study areas were purchased and subjected to post mortem examination performed by an experienced veterinarian. The selection criteria for these purchased animals were based on strong PPD response, unproductive animal, and willingness of farmer to sell the animal. The pathology scoring system was based on the semi-quantitative procedure developed by Vordermeier et al. [21]. Each lobe of the lung was inspected externally and then sliced into 2 cm-thick slices to facilitate detection of any typical TB lesion. Similarly, liver, spleen, and kidney as well as other lymph nodes (mandibular, medial retropharyngeal, bronchial, mediastinal, and mesenteric) were sliced into thin sections and inspected for the presence of typical TB lesions. The severity scores of the gross lesions and the pathology scores for lymph nodes, lungs, and other organs were added up to determine the total pathology score per animal. Furthermore, lesion type (caseous or calcified) and TB stage (localised or generalized) were documented.

### Sample collection, processing and culturing of mycobacteria

During post-mortem, up to seven suspected visible TB-like lesions were collected per animal in individual 50 ml sterile universal tubes (containing sterile PBS buffer) and transported at 4°C to the AHRI for further processing. At AHRI, samples were stored at 4°C when processed within 48 hours or at −20°C if processed later than two days after sample collection. All samples were processed and cultured for mycobacteria as previously described [7]. Samples were inoculated on three different media, including two Löwenstein-Jensen (LJ) media (supplemented with either glycerol or pyruvate) and a modified Middlebrook 7H11 medium optimised for the culture of *M. bovis* [22].

### Identification and molecular typing of mycobacteria

Bacterial colonies from culture positive samples were Ziehl-Neelsen stained according to standardized protocol to identify Acid-Fast Bacilli (AFB). Slants with no growth at week 8 were considered negative. Heat-killed cells of each AFB positive isolate were prepared by mixing ∼2 loopfuls of cells (∼20 µl cell pellet) in 200 µl dH_2_O followed by incubation at 80°C for 1 hour and then stored at 4°C until used for molecular typing. AFB positive isolates were also prepared as 20% glycerol stocks and stored at −80°C. Heat-killed AFB positive isolates were investigated by multiplex PCR for the presence or absence of RD4 [23], a chromosomal deletion characteristic of *M. bovis*. PCR amplification for RD4 typing was as follows: reactions were performed in a total volume of 20 µl consisting of 10 µl HotStarTaq Master Mix (Qiagen, United Kingdom), 7.1 µl distilled H_2_O, 0.3 µl of each oligonucleotide primer (100 µM), and 2 µl DNA template (heat-killed cells, see above). Isolates genetically typed as *M. bovis* were spoligotyped as previously described [24]. Selected *M. bovis* isolates were typed for 24-loci MIRU-VNTR by Genoscreen, France [25], [26].

### Data analysis

All collected field and laboratory data were double entered into Microsoft Access database. Data validation was done with EpiInfo software (“Data compare” package). Data analysis was performed with STATA Version 10.1 (StataCorp, Texas, USA). We used logistic regression models for the analysis of BTB prevalence and the univariate analysis of parameters associated with positive farms. A GEE model (Generalized Estimating Equations) with binary outcome was used to analyse potential risk factors for individual reactors, in which farms were used as random effect and farm size as fixed effect with adjusted odds ratio (OR). In both cases, results were presented with 95% confidence interval for OR and p-values.

## Results

### Study areas and farm recruitment

As shown in Figure 1, six study areas were chosen in the region of Addis Ababa in central Ethiopia; these were Addis Ababa, Sendafa, Holeta, Sululta, Debre Zeit and Sebeta. A total of 88 farms were included in the study, with a distribution of 14–16 farms per study area (Table 1). At least five small farms (1–10 cattle) and five medium size farms (11–50 cattle) were included from each study area, while the number of large dairy farms (>50 cattle) varied between two and five due to limited large herds in the Holeta (3), Sebeta (2), and Sendafa (2) study areas.

**Table 1:** Individual and herd prevalence of BTB based on CIDT and individual PPD-A response, both stratified by farm size and study site.

Farm size		Addis	Debre	Sebeta	Holeta	Sululta	Sendafa	Total
		Ababa	Zeit					
Small#	Farm number	6	6	5	6	5	6	34
	Animal number	40	25	37	36	34	46	218
	Indiv. prev. (%)	0	16	16.2	13.9	11.8	2.2	9.2
	Herd prev. (%)	0	33	40	16.7	40	17	23.5
	PPD-A resp. (%)*	0 (0)	11 (0)	2.7 (0)	0 (0)	6.2 (0)	2.2 (0)	3.2 (0)
Medium#	Farm number	5	5	7	6	5	5	33
	Animal number	187	106	177	181	154	137	942
	Indiv. prev. (%)	0.5	20.7	38.4	14.9	47.4	1.5	20.5
	Herd prev. (%)	20	100	71	67	80	40	63.6
	PPD-A resp. (%)*	3.2 (0.5)	1.9 (0)	8.5 (1.1)	2.8 (0)	22.7 (0.6)	0.7 (0)	6.8 (0.4)
Large#	Farm number	5	5	2	2	4	3	21
	Animal number	531	518	235	144	210	158	1796
	Indiv. prev. (%)	36.2	23.5	72.8	1.4	53.8	89.9	41.3
	Herd prev. (%)	80	100	100	100	100	67	90.5
	PPD-A resp. (%)*	17.1 (0.2)	11 (1.3)	11.1 (0)	9 (0.7)	10 (0.5)	24.7 (0.6)	13.8 (0.6)

# Farm size definition: Small, 1–10 animals; Medium, 11–50 animals; Large, >50 animals.

* The percent of animals with response to PPD-A alone [(A2-A1)>4 mm] regardless of the PPD-B response. In bracket, percentage of animals with a skin reaction defined as [(A2-A1) – (B2-B1)>4 mm].

### Dairy farm management

The vast majority (95%) of the investigated farms were privately owned while the remaining 5% were owned by a cooperative or the government. Seventeen of the 88 farms were owned by women. In our survey 80 out of these 88 farms answered our questionnaire (Questionnaire S1). These 80 farms were intensive dairy farms with similar husbandry practice and nearly all cattle herds were kept in indoor barns; only three small herds shared the same house as the owners and one farm kept its animals outdoors. Herds were strictly kept separate from others at 78 out of 80 farms while owners of the remaining two farms allowed mixing with animals from other farms. Sanitation status was assessed for the barns on each farm. The sanitation was mostly poor (20%) to medium (65%), while only 12 farms (15%) showed good sanitation standards. All farms included in this study used mainly their own bull or artificial insemination for restocking the herd. However, about 1/3 of the farms purchased animals during 2008 and 2009 and they did so within the area where this study was conducted (Figure 1). Fifty of the farm owners sold cattle during these two years of which 13 found a buyer in other locations of Ethiopia (outside the described study area) such as Tigray in the north, Ambo and Jimma in the west, and Nazareth, Hawassa, Woleita, Hosana, and Shashemene southwards of Addis Ababa. More than half of the farms that sold animals to regions outside the study area were medium-sized farms, whereas only ∼1/4 of both small and large farms had such behaviour. Animals were sold particularly when they showed low productivity (N = 50/80; 62%). Other motives for selling animals included being old (5%), weak and emaciated (5%), or being diseased (6.3%). Seven owners (8.9%) reported to have sold while still at high productivity.

### Trading and consumption of milk

The vast majority of milk produced in the investigated farms was either sold (N = 36/80; 45%), or partly sold and partly used for own consumption (50%). The remaining 5% of the farms produced milk for their own consumption only. Thirty-five farms sold their milk to processing plants, 23 to cafés and restaurants, 19 to intermediate caterers, and 16 directly to private individuals.

The number of people living on a farm varied between one and 53. However, the majority (81%) of farms accommodated less than ten people while half had only between one and four people living on the farm. The majority (86%) of the farm owners did not drink raw milk and 71% knew that BTB is an animal and a zoonotic disease.

### Tuberculin testing

Over a period of 13 months (January 2009 to January 2010), a total of 2956 cattle from 88 dairy farms were tuberculin tested by CIDT. The vast majority (94%) of these cattle were female and either of Holstein-Frisian breed (37%) or cross breeds thereof (62%) with local zebu cattle (Table 2). The remaining tested animals were of either zebu or Jersey breed, confined to a few farms in Holeta, Sendafa, Sebeta, and Debre Zeit.

**Table 2:** Univariate analysis of risk factors for having positive reactors using a GEE model with binary outcome.

Variable	Category	Animal No.*	BTB reactors*	OR (95%CI)	p-value
Herd size	Small	216 (7.3)	20 (2.1)		
	Medium	942 (31.9)	193 (20.2)	2.5 (0.91; 6.85)	0.07
	Large	1796 (60.8)	742 (77.7)	5.9 (2.06; 16.84)	0.001
Breed	Crossbreed	1837 (62.8)	351 (37.2)		
	Holstein	1011 (34.6)	582 (61.8)	1.2 (0.69; 2.23)	0.47
	Zebu	45 (1.5)	3 (0.3)	1.1 (0.89; 1.33)	0.39
	Jersey	33 (1)	7 (0.7)	1.1 (0.73; 1.71)	0.59
Sex	Female	2783 (94)	919 (96.3)		
	Male	170 (6)	35 (3.7)	0.9 (0.76; 1.23)	0.8
Body condition score	Poor	212 (7.2)	62 (7)		
	Medium	1454 (49.5)	409 (43)	0.8 (0.57; 1.06)	0.1
	Good	1272 (43.3)	481 (50)	0.8 (0.58; 1.13)	0.2
Age class	<12 months	139 (5.2)	27 (3)		
	≥12 mo - 3 yrs	823 (31)	225 (24.3)	2.2 (0.91; 5.51)	0.07
	>3 yrs - 10 yrs	1642 (61.8)	656 (71)	3.6 (1.43; 9.30)	0.007
	>10 yrs	54 (2)	16 (1.7)	4.1 (1.59; 10.56)	0.003
PPD-A status	Negative	2636 (89.2)	755 (79)		
	Positive	318 (10.8)	200 (21)	1.6 (1.01; 2.60)	0.04

* A number in brackets shows percentage of animals recorded in a category of one variable. The total number of animals recorded for in one variable may vary as some data are missing.

The result of the tuberculin testing is presented in Table 1 as prevalence per study area and herd size. The CIDT result shows that BTB was prevalent in all six study areas. It also demonstrates that the prevalence increased with farm size category. The average individual prevalence of small, medium, and large farms was 9.2%, 20.5%, and 41.3%, respectively, and a similar trend was seen in herd prevalence with the extreme of over 90% infected herds among the large farms (Table 1). The overall number of herds with at least one reactor animal was 48 (55%); however, 38 farms (43%) had ≥10% of its animals infected (Figure 1B).

More detailed analysis of small and medium sized farms indicated that the areas of Addis Ababa and Sendafa (Figure 1) had the lowest individual animal prevalence; only one out of 86 tested animals from small farms in those two areas combined was a reactor, and in the medium-sized farms only three out of 324 animals were positive according to the CIDT. Small and medium farms tested in the remaining four study areas had significantly higher individual prevalence, ranging between 12–16% in small farms while it varied more in medium farms (between 15–47.4%); elevated prevalence was seen in medium farms in Sebeta and Sululta. The distribution of tuberculin reactors between farms is reflected in the herd prevalence (Table 1). More than 1/3 of small farms and over 2/3 of medium farms were infected in Debre Zeit, Sebeta, and Sululta. The latter was valid also for the medium farms in Holeta. We conclude that BTB was widely distributed among the tested small and medium farms in at least Debre Zeit, Sebeta, and Sululta.

The results obtained from the analysis of large dairy farms showed a somewhat different picture. The individual BTB prevalence in any tested large farm varied from 0% and up to 93%. By study area, Holeta showed the lowest prevalence with only two CIDT positive animals between the two large farms tested. The individual prevalence in all other study areas varied between 23.5–90%, (Table 1). Overall, 18 out of the 21 large farms were infected with BTB (overall herd prevalence: 85.7%) of which 11 had an animal prevalence of over 30%. The crude overall individual prevalence was 32.3% (30.6%–34.0%) (not considering stratification of farm size or study sites).

### Sensitisation to environmental mycobacteria

Although the CIDT is used to detect BTB infection with high specificity, reactivity to the avian PPD indicates infection with or exposure to species of the *Mycobacterium avium* complex (MAC) or other environmental mycobacteria. In this study 318 out of the 2956 tested animals (∼11%) reacted positive to PPD-A. The PPD-A response was lowest in Holeta (5%; 95%CI: 3.1; 7.8) and highest in Sululta (14.6%; 95%CI: 11.5; 18.5) (Table 1), regardless of farm size. However, 200 out of the 318 PPD-A positive animals reacted positive also to PPD-B (Table 2) and the PPD-A responses could be down to cross reactivity between *M. bovis* antigens and antigens from environmental mycobacteria. Interestingly, PPD-A responders with a stronger response than to PPD-B were few (between 0 and 1.3%; Table 1).

### Factors associated with BTB

The univariate analysis (Table 2) showed that reactor animals differed statistically according to age categories. Adult animals between 3 and 10 years old were over threefold more at risk of being tuberculin positive (with adults older than 10 years fourfold more at risk) as compared to young animals (OR = 3.6, p-value = 0.007 and OR = 4.1, p-value = 0.003, respectively). Medium and large farms had a significant risk increase of having positive reactors (OR = 2.5 and OR = 5.9 respectively, p-value: 0.001). Positive PPD-A reactors had an OR of 1.6 for being BTB reactors (p-value: 0.04).

Table 3 shows the univariate analysis of parameters potentially affecting the BTB herd status: Herd size and education level of the owners were factors associated with having positive farms; medium herds had a statistically significant fivefold increase of tuberculin positivity (OR = 5.4; p-value: 0.002) compared to small herds. Higher education of owner was linked with BTB positive farms (secondary school with OR = 4.8, p-value: 0.04 and diploma/degree with OR = 4.5, p-value: 0.05). The majority of the owners (65.4%) had an education above junior secondary school (secondary school, diploma, degree). However, 87.5% of the owners having large farms had gone through secondary school and/or higher education (diploma, degree). On the other hand 50% of the owners with no education at all or primary school attendance governed over small farms. When stratified by farm size, there was no statistical difference between level of education and BTB farm status (data not shown).

**Table 3:** Univariate analysis for potential risk factors being a positive farm (logistic regression with likelihood ratio test).

Variable	Category	Farm No.	BTB+ farms	OR (95% CI)	p-value
Herd size	Small	32 (40.5)	9 (19.6)		
	Medium	31 (39.2)	21 (45.6)	5.4 (1.82; 15.76)	0.002
	Large	16 (20.3)	16 (34.8)	-	-
Owner education level	No school	11 (14)	3 (6.7)		
	Primary school	9 (11.6)	5 (11)	3.3 (0.51; 21.58)	0.2
	Secondary school	31 (39.8)	20 (44.5)	4.8 (1.06; 22.10)	0.04
	Diploma and degrees	27 (34.6)	17 (37.8)	4.5 (0.97; 21.14)	0.05
BTB knowledge	Yes	57 (71.2)	36 (76.6)		
	No	23 (28.8)	11 (23.4)	0.5 (0.20; 1.42)	0.21
Owner gender	Male	62 (78.5)	39 (84.8)		
	Female	17 (21.5)	7 (15.2)	0.4 (0.13; 1.23)	0.11
Source of cattle purchase	Dairy farms	55 (71.4)	31 (67.4)		
	Markets, unknown origin	12 (15.6)	8 (17.4)	1.5 (0.41; 5.75)	0.51
	Gift	2 (2.6)	1 (2.2)	0.8 (0.04; 13.02)	0.85
	Research stations	5 (6.5)	3 (6.5)	1.2 (0.17; 7.51)	0.87
	Dairy farms and markets	3 (3.9)	3 (6.5)	-	-

There was no significant difference in BTB status amongst farms with different sanitation status and amongst the type of cattle housing (data not shown).

### Ante- and post-mortem investigation

**Figure 2:**
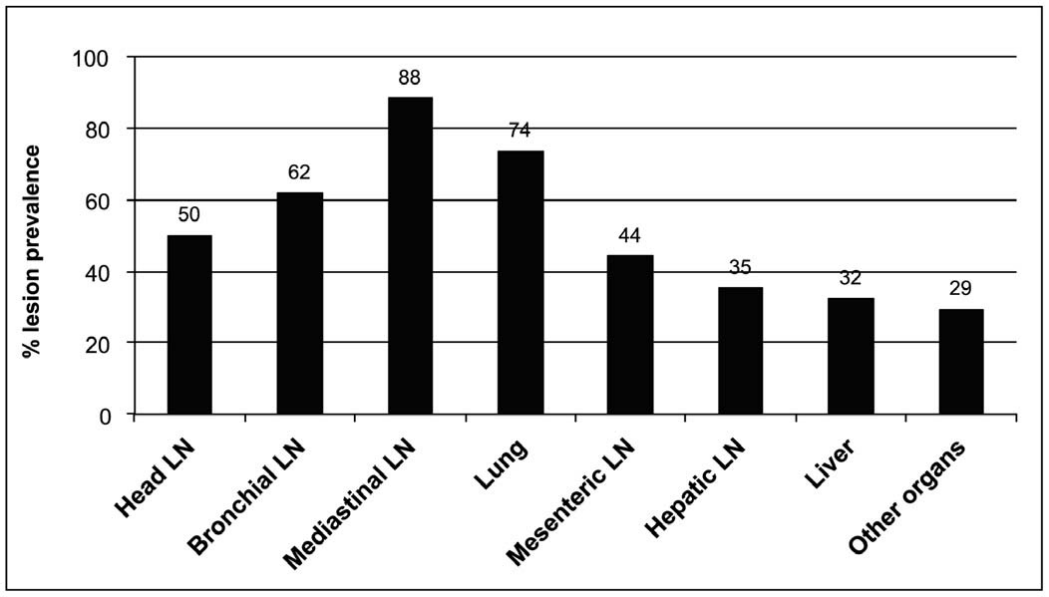
Organ association of suspect TB lesions in 34 cattle with visible lesions (LN, lymph node).

Over 90% of all investigated animals had medium or good body condition. However, no significant difference of the body condition score was observed between tuberculin positive and negative cattle (Table 2). To further confirm that the recorded high rates of BTB were due to *M. bovis* and to get a better understanding of the population structure of the causative agent, we purchased and slaughtered a minimum of three PPD positive animals per study area. In total 36 cattle from 19 farms (up to three animals per farm) were purchased and investigated by post-mortem and each animal was given a pathology score based on disease progression (Table 4). A trend of inverted correlation between pathology score and PPD response was observed; animals with strong response to the tuberculin test had in general a lower than average pathology score. Suspect TB lesions from up to seven infected sites per animal were collected from 34 animals. In addition, samples were taken from mediastinal and bronchial lymph nodes from two animals with non-visible lesions. Thirteen out of the 34 (32%) animals with lesions had generalised disease while the remaining 21 had lesions localised to one or a few infection sites. Figure 2 shows the distribution of lesions found in the 34 animals. Visible lesions were most frequent in mediastinal lymph nodes (N = 30; 88%), lungs (74%), bronchial lymph nodes (62%), and head lymph nodes (50%), but prevalent also in mesenteric and hepatic lymph nodes, and in liver. Calcification was the most commonly observed stage of lesion (Table 4).

**Table 4:** Post-mortem results of 36 animals from 19 farms.

Study area	Farm ID:	CIDT	PM	Type of TB	Type of lesion	AFB	Strain ID	*M. bovis*	24-loci MIRU-VNTR type*
	Animal ID	(Δmm)	score					Spoligotype	
Addis Ababa	1:A	24	4	localized	caseous	Yes	BTB-2269	SB0134	35223 40554 21415 24222 2XXX
Addis Ababa	1:B	26	15	localized	calcified	No	-	-	-
Addis Ababa	2:A	22	23	generalized	calcified/caseous	Yes	BTB-2850	SB1477	36223 32454 21445 24222 3213
Addis Ababa	2:B	18	23	generalized	calcified	Yes	BTB-2840	SB1176	35223 32554 21435 24222 3323
Holeta	3:A	30	10	generalized	caseous	Yes	BTB-2352	SB1176	35223 32554 21435 24222 3323
Holeta	3:B	26	0	-	-	No	-	-	-
Holeta	3:C	12	29	generalized	calcified	Yes	BTB-2400	SB1176	35223 32554 21435 24222 3323
Holeta	4:A	29	5	generalized	calcified	Yes	BTB-2349	SB0134	-
Holeta	5:A	11	20	localized	calcified	Yes	BTB-2350	SB0134	35223 40554 21415 24222 2332
Sululta	6:A	16	14	localized	calcified/caseous	Yes	BTB-2762	SB0133	23223 32454 21425 24222 3323
Sululta	6:B	18	21	generalized	calcified	Yes	BTB-2822	SB0133	23X23 32454 21425 24222 3323
Sululta	7:A	15	46	generalized	calcified	Yes	BTB-2466	SB1176	35223 32554 21435 24222 3323
Sululta	7:B	28	12	localized	calcified	Yes	BTB-2785	SB1176	35223 32554 21435 24222 3323
Sululta	7:C	14	43	generalized	calcified	Yes	BTB-2469	SB1176	35223 32554 21435 24222 3323
Sululta	8:A	38	5	localized	calcified	Yes	BTB-2465	SB1176	35223 32554 21435 24222 3323
Sululta	8:B	24	1	localized	calcified	Yes	BTB-2461	SB0134	35223 40554 21415 24222 2332
Sendafa	9:A	21	14	generalized	calcified/caseous	Yes	BTB-2847	SB0134	35223 40554 21415 24222 2332
Sendafa	9:B	13	8	localized	caseous	Yes	BTB-2849	SB0134	35223 40554 21415 24222 2332
Sendafa	10:A	17	2	localized	caseous	Yes	BTB-2862	SB0133	-
Sendafa	10:B	41	4	localized	calcified/caseous	Yes	BTB-2861	SB0133	23223 32454 2X425 24222 3X23
Debre Zeit	11:A	17	20	generalized	calcified	Yes	BTB-2266	SB1176	-
Debre Zeit	11:B	16	19	generalized	calcified	Yes	BTB-2261	SB0134	35223 40554 21415 24222 2332
Debre Zeit	12:A	4	9	localized	calcified	Yes	BTB-2260	SB1176	35223 32552 21435 24222 3323
Debre Zeit	13:A	23	19	generalized	calcified	No	-	-	-
Debre Zeit	13:B	11	8	localized	calcified	Yes	BTB-2257	SB0133	23223 32454 21425 24222 3323
Sebeta	14:A	6	2	localized	calcified	No	-	-	-
Sebeta	14:B	20	12	localized	caseous	Yes	BTB-2813	SB1176	3522X 32554 2X435 24X22 3323
Sebeta	15:A	18	37	generalized	calcified	Yes	BTB-2803	SB1176	35223 32552 21435 24222 3323
Sebeta	15:B	18	6	localized	caseous	Yes	BTB-2805	SB1176	35223 32552 21435 24222 3323
Sebeta	16:A	20	0	-	-	No	-	-	-
Sebeta	16:B	22	4	localized	calcified	Yes	BTB-2768	SB1176	-
Sebeta	17:A	21	8	localized	calcified	Yes	BTB-2838	SB0134	35223 40554 21415 24222 2332
Sebeta	17:B	16	8	localized	calcified/caseous	Yes	BTB-2765	SB0134	35223 40554 21415 24222 2332
Sebeta	18:A	13	6	localized	caseous	Yes	BTB-2757	SB0134	35223 40554 21415 24222 2332
Sebeta	19:A	22	9	localized	calcified	Yes	BTB-2773	SB0134	35223 40554 21415 24222 2332
Sebeta	19:B	20	13	localized	calcified/caseous	Yes	BTB-2766	SB0134	35223 40554 21415 24222 2332

The average CIDT value and post-mortem score [21] were 20 mm and 13, respectively.

* Consecutive order of the 24-loci MIRU-VNTR: 2165; 2461; 577; 580; 3192; 154; 960; 1644; 2059; 2531; 2687; 2996; 3007; 4348; 802; 424; 1955; 2163b; 2347; 2401;3171; 3690; 4052; 4156; X = Typing failed.

### Isolation and molecular typing of mycobacteria

Mycobacterial culturing on selected media yielded in total 107 AFB isolates from samples corresponding to 31 animals with visible lesion. No AFB were cultivated from the remaining three animals with suspect TB lesions or from samples of the two animals with non-visible lesions (Table 4).

Heat-killed cells of 67 AFB isolates originating from 31 animals were enrolled in a typing scheme initiated by RD4 deletion typing. The result showed that all isolates could be identified as *M. bovis* as they had the RD4 region deleted [23]. Spoligotyping was then employed to further differentiate these isolates and the outcome is shown in Table 4. Overall, four different spoligotype patterns were recognized among these *M. bovis* isolates and defined as of type SB0134, SB1176, SB0133, and SB1477 at the international spoligotyping database www.mbovis.org. None of the 31 animals were found to be infected with more than one *M. bovis* spoligotype suggesting no dual infection among these animals. On the other hand, *M. bovis* isolates of two different spoligotype patterns were collected from three farms (out of 19). The geographical distribution of these four spoligotypes suggested three main clusters; SB0134 was the most dispersed and found in all six study areas, SB1176 was represented in all areas but Sendafa, while SB0133 was only found in Sululta, Sendafa and Debre Zeit. The one isolate of type SB1477 was collected in a farm from Addis Ababa.

In attempts to find possible transmission links between dairy farms included in this study we performed 24-loci MIRU-VNTR typing to further discriminate the collected *M. bovis* isolates. MIRU-VNTR results were generated for 27 isolates of the four different spoligotypes (Table 4). All 11 isolates of type SB0134 had the same genotype (spoligotype plus MIRU-VNTR type) and that was also the case for four isolates of SB0133. Only the collected isolates of type SB1176 showed diversity; two different genotypes were identified among 11 isolates, however the two types differed in only one out of the 24 loci (Locus 2531; Table 4). Overall, this genotyping result suggested very low diversity within each spoligotype cluster among the collected strains.

In a previous study on BTB in Ethiopia [7] we isolated and identified *M. bovis* from several abattoirs in the country. Here we performed 24-loci MIRU-VNTR typing of 15 *M. bovis* isolates from that study with spoligotypes SB0133, SB1176, SB0134, the three most frequent types isolated in the present study (Table 4) with the aim to investigate genotype diversity among strains collected from different parts of Ethiopia. Table 5 summarises the different *M. bovis* genotypes and their frequency from each collection site. Two distinct MIRU-VNTR features were seen among strains within spoligotype SB0133 as well as within SB1176, while strains compared within SB0134 showed no diversity.

**Table 5:** Geographical diversity of *Mycobacterium bovis* genotypes in Ethiopia.

Spoligotype	Region	Collection site	24 loci MIRU-VNTR type*	Frequency
SB0133	North Ethiopia	Woldiya	23223 32454 21425 24222 3323	1
	Central Ethiopia	This study	23223 32454 21425 24222 3323	3
	Central Ethiopia	Addis Ababa	23223 32454 214**3**5 24222 3323	1
	South-East Ethiopia	Negelle	23223 32454 21425 24222 3**2**23	1
	South-East Ethiopia	Negelle	23223 32454 21425 24222 3323	2
	South Ethiopia	Jinka	**36**223 **2**2**53**4 214**36** 24222 **4**323	1
	South Ethiopia	Jinka	**36**223 **2**2**54**4 214**36** 24222 **4**323	4
	West Ethiopia	Ghimbi	**36**223 **2**2**54**4 214**36** 24222 **4**323	1
SB1176	North Ethiopia	Woldiya	35223 32554 2143**4** 24222 3323	2
	Central Ethiopia	This study	35223 32554 21435 24222 3323	8
	Central Ethiopia	This study	35223 3255**2** 21435 24222 3323	3
	South Ethiopia	Jinka	35223 3**3**5**7**4 **3**1**223** 24222 33**7**3	1
SB0134	North Ethiopia	Gondar	35223 40554 21415 24222 2332	1
	Central Ethiopia	This study	35223 40554 21415 24222 2332	11

Repeats of locus highlighted in bold diverge between isolates of the same spoligotype. All collection sites are shown in Figure 1.

* Consecutive order of the 24-loci MIRU-VNTR: 2165; 2461; 577; 580; 3192; 154; 960; 1644; 2059; 2531; 2687; 2996; 3007; 4348; 802; 424; 1955; 2163b; 2347; 2401; 3171; 3690; 4052; 4156.

## Discussion

With the recent shift towards larger intensive farming systems, mainly in urban areas, it is clear that the farming practices are becoming more favourable for BTB transmission. In this study we explored the prevalence of BTB in cattle in intensively managed dairy farms located in and around Addis Ababa and that supply milk to the capital. Based on the CIDT test, all six surveyed areas (Figure 1) recorded a high rate of the disease and we concluded that, overall, approximately one third of the tested animals and more than half of the investigated herds were infected with BTB (if stratification or random effect is not taken into account). However, the prevalence fluctuated significantly between the three investigated farm size categories. Both the overall individual and overall herd prevalence of medium and large farms were double and fourfold, respectively, as compared to the corresponding numbers of small farms (Table 1). Since as many as 20 out of 21 large herds (>50 animals/herd) were infected, we concluded that BTB had a strong-hold in dairy farms in this investigated region of central Ethiopia.

### Risk factors

Several past and recent studies have shown that susceptibility to BTB can vary between cattle breeds [12], [27] with suggestions that cattle of *Bos indicus* (Zebu) breeds are more resistant to BTB than *Bos taurus* (European breeds). The vast majority of the dairy cattle included in this study were cross breeds (62%) and Holstein-Frisian (34%), however the BTB prevalence did not differ significantly between these two categories (Table 2). The number of cattle recorded as Jersey and Zebu breeds in this study were too few to make relevant comparisons on susceptibility. On the other hand, Ameni et al. [28] have demonstrated that animal husbandry conditions are a major influence on the prevalence of BTB. Cattle kept under high-intensity conditions showed significantly higher skin-test prevalence as compared to cattle kept under extensive conditions. Intensification, stressed animals, and overcrowding are all possible explanations for such relationship. The main routes of BTB transmission are through aerosol as gross lesions usually involve the lungs and thoracic lymph nodes [29], and therefore BTB transmission benefits from overcrowded herds. The post-mortem data collected in this study support this as most animals had TB lesions in lungs and/or lung associated lymph nodes (Figure 2). Once an animal is infected with BTB, it usually takes several months or longer for this chronic disease to develop clinical signs. It is also likely that infected cattle, similarly to humans, enter an asymptomatic phase after infection with *M. bovis*, in which no clinical signs are developed for years [30]. Our ante- and post-mortem investigation also confirmed this common attribute of BTB. The body condition scoring of the nearly 3,000 animals suggested no significant differences between tuberculin reactors and non-reactors (Table 2) and a poor correlation was also seen among the 36 slaughtered animals; despite that the majority of these animals were highly diseased with severe pathology, little or no clinical signs typical for BTB were observed. Thus, if BTB gets into a herd where no diagnostic measure is in place (such as CIDT), then it can be difficult to detect infected animals on a visual basis solely and to prevent further transmission.

Most owners of medium and large farms had higher educational level while illiterate farmers tended to have smaller farms. Although this leads to an association in our dataset between higher education and higher prevalence of BTB in the herds (Table 3), this is more likely to be an expression of higher educated people engaging in dairy farm business as an investment, hence larger farms being associated with higher education.

Farmers participating in the current study said that the main means of restocking their herd was through artificial insemination. However, frequently animals were purchased or sold between farms within the study areas. Trading of animals beyond the study area occurred too (mainly by medium sized farms) but seemed unidirectional; dairy cattle were not purchased but only sold to other regions across Ethiopia. The response from farmers in this survey also suggested that some animals were sold because they were weak or diseased. As no official test-and-movement policy is in place, selling of infected animals from this highly affected region of Ethiopia may consequently contribute to spreading BTB to other parts of the country.

### The causative agent

The mycobacterial strains isolated from cattle in this study were solely *M. bovis* and the CIDT test was confirmed by culture in 86% of the slaughtered cattle. This culturing yield can be compared with a 90% yield normally seen in slaughterhouse cases in the UK from animals with visible lesions and of which the vast majority were caused by *M. bovis* [31]. No strain of *M. tuberculosis* was isolated in our study in contrast to several other Ethiopian studies [7], [13], [32]. It can be argued though that only ∼3% of all cattle that were tuberculin positive in this study were investigated by post-mortem, leaving out a large group of animals that could have been infected with *M. tuberculosis* as CIDT testing do not distinguish between *M. tuberculosis* and *M. bovis*. However, the dairy cattle of this study were managed under intensive husbandry systems with animals staying in separate barns, as compared to management systems common in the rural areas where domestic animals often sleep in the same house as people. It has previously been suggested that human-to-cattle transmission of *M. tuberculosis* is possible in settings where farm members in high-burden TB areas are in close contact with their animals [32].

Molecular typing of the 31 *M. bovis* strains isolated from the slaughtered animals identified four different spoligotype patterns which previously have been shown as common in several regions of Ethiopia [7]. Three of these four spoligotypes (SB1176, SB1477, and SB0133) carry a specific spoligotype feature (spacers 4–7 missing) typical for members of a clonal complex identified as *M. bovis* African 2 (Af2) so far only found in East Africa [33]. The fourth spoligotype (SB0134) do not belong to the Af2 clonal complex and is therefore likely to have a different epidemiological history. *M. bovis* isolates with spoligotype SB0134 has been found also in Europe. However, the phylogenetic relationship between the African and European strains of SB0134 is not known. Future chromosomal sequencing may shed light over their relationship.

Further discrimination of the *M. bovis* isolates performed by MIRU-VNTR typing suggested low diversity within each isolated spoligotype. This is in agreement with other studies as in high prevalence settings the genetic diversity of sampled strains is usually low, reflecting on-going local transmission events (due to high transmission rate) [34]. However, in comparison with genotypes of *M. bovis* strains that were collected from other parts of Ethiopia about 2–3 years earlier [7], some variations were observed and possible epidemiological links between specific regions of the country can be suggested (Table 5). Two distinct MIRU-VNTR features were seen within spoligotype SB0133 of which the one identified in central Ethiopia (this study) was also seen in sites along the Rift Valley, from Woldiya in the north to Negelle in the south. The other genotype of SB0133 was isolated from Jinka (south-west Ethiopia) and Ghimbi (west Ethiopia). Similarly, the single strain of type SB1176 collected in Jinka deviated in the MIRU-VNTR pattern from those seen in Woldiya and in central Ethiopia. However, no genotype diversity was seen between the one isolate of SB0134 from Gondar (North-west) when compared to the 11 *M. bovis* strains of SB0134 isolated in central Ethiopia (this study). Overall, based on these molecular typing results of our *M. bovis* collection, epidemiological links can be suggested for strains collected within the North/Central Ethiopian highlands and through the rift valley down to Negelle, while strains isolated from Jinka and Ghimbi in the south/west of Ethiopia diverged. This may reflect on past and ongoing cattle movements and trading patterns in the country. The low strain frequency from some collection sites means that these results should be interpreted with care. Nevertheless, this genotype comparison gives an indication of the diversity and the epidemiology of *M. bovis* in Ethiopia. Future studies may give better clarification on this subject. The 24-loci MIRU-VNTR typing employed for this study showed no diversity in at least 14 of the 24 loci (variation seen between spoligotypes; Table 5). This is in agreement with other studies [35], which has shown that VNTR typing of only a selective set of loci is informative for generating a better resolution between *M. bovis* isolates. This suggests that any future MIRU-VNTR typing of *M. bovis* strains from Ethiopia could consider including only loci that show diversity in its local strain population, however, a more comprehensive evaluation of what loci to select is recommended.

### Reasons for controlling BTB

At least three important reasons for controlling BTB should be considered: animal welfare, the financial burden to farmers with diseased animals, and the risk of zoonotic transmission. This chronic disease can take years to develop. Animals infected with BTB can therefore slowly develop symptoms and may, if not carefully observed by animal keepers, suffer unnecessarily. Depending on which organ or associated lymph node is affected, animals can display a wide range of symptoms e.g. coughing, dyspnoea, gastrointestinal problems, bone deformation, and emaciation [36].

Few thorough studies have been conducted to investigate loss of productivity due to BTB, but it is probable that there is a productivity impact in animals with BTB due to the nature of the disease and thus this is likely linked to economical losses [37]. It has previously been suggested that reduced milk production, the food value of the carcass, and reproduction (or fertility) are factors that affects the animal productivity [38].

The zoonotic risk of BTB is often associated with consumption (ingestion) of dairy products based on unpasteurised milk infected with *M. bovis*. However, aerosol transmission (inhalation) from cattle-to-human should also be considered as a potential risk factor. Ethiopian milk consumers generally prefer raw milk (as compared to treated milk) because of its taste, availability and lower price. Therefore the demand for fresh whole milk in rural areas is and will probably remain high, and in general, the demand from these consumers is likely to be satisfied by home production or by purchasing from neighbouring producers. On the other hand, the markets for surplus milk are mainly found in the urban population, of which currently ∼65% is accounted for by the capital Addis Ababa and its neighbouring districts [39].

The annual total milk supply to Addis Ababa has been estimated at 65 million litres and the major sources are from small private farms and smallholder urban dairies in and around the city that own upgraded breeds [40]. These over 5,000 dairy farms produce around 35 million litres of milk per year. Grossly 10% is used to feed calves, 10% is used for home consumption, 8% is processed into butter and cheese, while the remaining ∼2/3 is sold to the market as un-boiled or unpasteurised milk [41]. Other important sources of milk to the capital city are medium to large dairy plants run privately or as enterprises/cooperatives. Today there are around eight major dairy plants in central Ethiopia with a total production capacity of around 100,000 litres per day (∼35 million litres per year) (survey by Alehegne Wubete, unpublished), but the actual yearly production is likely to be less as animal product consumption can vary through the year due to cultural and religious habits such as fasting. These plants collect milk from farmers within a 150 km radius around Addis Ababa and provide standardized pasteurized milk in plastic sachets to the markets [39]. Our survey suggests that many dairy farms from the investigated area sell their milk to restaurants and catering companies, but it is difficult to estimate how much of that milk is pasteurised or boiled before consumption. Overall, the available information suggests that a significant volume of milk may be distributed without being pasteurised.

In a recent Ethiopian study of the rate of *M. bovis* causing TB in humans [42] we reported that the rate of TB in humans due to *M. bovis* is currently below 1%. However, we concluded that areas where BTB prevalence in cattle is high (such as the “hot-spot” area of central Ethiopia shown in this study) could generate an increase of BTB in the human population in such areas. It is important to note that existing human TB control methods relying on the directly observed treatment short course (DOTS) strategy will not directly impact on BTB transmission. The high rate of BTB in the dairy farms of this study is therefore likely to pose a serious risk to public health and deserves a targeted intervention as early as possible.

### Conclusion and Control strategies

This study suggests that the overall prevalence of BTB in intensive dairy farms in Addis Ababa and its surroundings is very high and requires urgent intervention to control the disease. To comprehensively control BTB regardless of the method chosen, a robust system of individual animal and herd identification is required. The powers to restrict movements to prevent disease dissemination and the ability to trace movements to catch up with translocation are essential components of an eradication strategy. Most developed countries where BTB is a problem have introduced a test-and-slaughter policy to control/eradicate the disease. The CIDT, although not 100% sensitive, is the standard test widely used in developed countries for this purpose. Most importantly, infected animals have to be detected and removed at an early stage to minimise further transmission. As such policy is considered costly it has rarely been implemented in developing countries. No compensation scheme for elimination of infected animals is currently in place in Ethiopia and due to financial constraints changes toward such policy might not be feasible in the near future. However, a test-and-segregation policy of tuberculin positive animals can be suggested and pursued and the milk of infected animals strictly and consequently pasteurized before selling and consumption. Additional interventions by the government authorities could be to encourage farmers to regularly test their animals for BTB and create incentives to keep their herds free from BTB. It could be compulsory for dairy farms that are supplying milk to the public to test their cattle for BTB, and create incentives to TB free herds – “good milk certificate” – that could lead to better economic values for farmers. Also dairy plants should be engaged and could e.g. pay a higher rate for milk from herds certified as being BTB free. Pasteurization at a bigger scale has to be promoted, either directly at farm level before selling the milk or at household level through boiling the milk before consumption (e.g. awareness campaigns).

As the dairy industry in Ethiopia has expanded in recent years and is expected to continue doing so, significant number of high productive exotic and cross breed animals are likely to be traded from the urban areas around the capital to the rural areas where dairy cattle numbers are still relatively low. Such movement is linked with increasing financial well-being of farmers and amelioration of artificial insemination technique/delivery. However, without any control strategy the risk of spreading BTB by such movements is high and may create new hot-spots of BTB in other parts of the country. Several recommendations can be made to minimize the risk of spreading the disease between farms and regions. First and most important is to spread knowledge about BTB and make farmers and people involved in trading of cattle aware of the risks involved in trading animals. On a smaller scale, our field work and specific training to the farmers aimed on this, informing them in how to handle cattle infected with BTB and other diseases, and encouraging them not to trade with diseased animals. Another way to decrease risk of transmission is to encourage farmers who are restocking by purchasing animals to request that the animals should be tested for BTB. The CIDT test is relatively inexpensive and would reduce the risk to the farmer of purchasing an infected animal. A possibility for Ethiopia to in part meet the demand for cattle of upgraded breeds could be to establish farms with dairy cattle free of BTB and from which farmers could restock their farms without the risk of introducing the disease into their herds. This might imply scaling up of existing artificial insemination centres to improve productivity in the national dairy herd and possibly purchase of new stocks of exotic breeds from abroad.

## Supporting Information

Questionnaire S1 (PDF)
