# Supplementary material for: High Prevalence of Bovine Tuberculosis in Dairy Cattle in Central Ethiopia: Implications for the Dairy Industry and Public Health
Source: PLoS One. 2012 Dec 28;7(12):e52851. doi: 10.1371/journal.pone.0052851 (PMC3532161; doi:10.1371/journal.pone.0052851)
Supplement: Questionnaire S1 — (PDF) [file pone.0052851.s001.pdf]

Following questionnaire was used in the study "High prevalence of Bovine Tuberculosis in Dairy cattle in Central Ethiopia: Implications for the Dairy Industry and Public Health" by Firdessa et al. This questionnaire has three parts dealing with (A) general information about the farms and their owners, (B) possible risk factors related to bovine tuberculosis, and (C) public health risks and awareness.

Survey performed by AHRI/NAHDIC

Questionnaire number: _______

Date of interview: ____/____/________

Interview performed by: __________________

Answering the questions is only depend on your good will and can be withdrawn at any time during the interview. Please answer the questions in absolute number/text or mark the number of the correct option(s).

A. Questions about the farm and its owner

1. General information

a. Dairy farm name: ____________________________

b. Name of the dairy farm owners (or animal attendant working in the farm not less than one year): ______________________________

c. Address: Region/city_______________District/Subcity_______________Kebelle_______

d. Age (years): |__|__|

e. Sex: |__|

f. 1. Male 2. Female

g. Educational status: |__|

1. Illiterate 2. Basic writing & reading 3. Primary (Grade 1 to 6)

4. Junior secondary (grade 7 to 8) 5. Secondary (Grade 9 to 12)

6. Diploma 7. Degree and above

h. How many people are living or working on the farm? |__|__|

2. How did the owner start the farm business (multiple options possible)?

a. Bought the enterprise 1. Yes |__| 2. No |__|

b. Bought cattle from other known dairy farms 1. Yes |__| 2. No |__|

c. Bought animals from market without knowing their origin 1. Yes |__| 2. No |__|

d. Gift 1. Yes |__| 2. No |__|

e. Other 1. Yes |__| 2. No |__| If yes, specify: _________________________

B. Questions related to possible risk factors for bovine tuberculosis

3. Type of house/barn: |__|

1. Indoor 2. Outdoor

3. None, but fenced 4. Cattle share house with the owners

4. Sanitary condition of the barn/house based on odors, waste drainage, cleanness of floor and animals, light source, and animal stocking: |__|

1. Poor 2. Medium (satisfactory condition) 3. Excellent

5. Ventilation status of the barn/house: |__|

1. Poor 2. Medium (satisfactory ventilation) 3. Excellent

6. The purpose of the Dairy farm: |__|

1. To produce dairy products for home consumption only
2. To produce dairy products for market only
3. To produce dairy products for market and home consumption

7. What is the total milk production on your farm per year? |__|__|__|__|__|__|__|Liters/year

8. What is the average milk production per cattle per year?

|__|__|__|__|__|__| Liters/year

9. To whom do you sell the milk (multiple options possible)?

1. To individual consumers 1. Yes |__| 2. No |__|
2. To processing plant 1. Yes |__| 2. No |__|
3. To intermediate cater 1. Yes |__| 2. No |__|
4. To restaurants/cafeteria 1. Yes |__| 2. No |__|
5. Other, 1. Yes |__| 2. No |__|specify: ____________________

10. How do you get replacement stock (multiple options possible)? |__|

1. My own farm by Artificial Insemination
2. Insemination by own bull
3. Purchasing from different cattle sources
4. Other, specify: __________________

11. From what area have you purchased cattle during 2008-09 (specify farm and woreda)?

____

12. To what area have you sold cattle during 2008-09 (specify farm and woreda)?

____

13. What type of cattle do you sell from your farm (multiple options possible)?

1. Weak / poor body condition 1. Yes |__| 2. No |__|
2. Diseased 1. Yes |__| 2. No |__|
3. Low productive 1. Yes |__| 2. No |__|
4. High productive 1. Yes |__| 2. No |__|
5. Other, Specify:_______________________________________

14. Are animals in your farm mixed with animals from other farms?

1. Yes |__| 2. No |__|

15. Have you in the last six months, had any animal in your herd with chronic cough/chronic body wastage? 1. Yes |__| 2. No |__|

16. Have cattle been tuberculin/PPD tested before on your farm?

1. Yes |__| 2. No |__|

- ❖ If yes, what happened with the cattle tested as positive (multiple options possible)? |__|
  1. It remained at the farm 1. Yes |__| 2. No |__|
  2. It was slaughtered 1. Yes |__| 2. No |__|
  3. It was sold 1. Yes |__| 2. No |__|

<u>C. Questions related to public health risk and awareness</u>

17. Do members of your family/farm drink raw milk regularly (once per month or more)?

1. Yes |__| 2. No |__|

18. Do you know that bovine tuberculosis is a cattle disease?

1. Yes |__| 2. No |__|

19. Do you know that bovine tuberculosis can be transmitted to man through raw milk/milk products consumption obtained from bovine tuberculosis infected cattle?

1. Yes |__| 2. No |__|

20. Do you know that bovine tuberculosis can be transmitted to man through raw meat consumption obtained from bovine tuberculosis infected cattle?

1. Yes |__| 2. No |__|

21. Have any of the people living/working on your farm had tuberculosis in the last two years?

1. Yes |__| 2. No |__|

- ❖ If anyone has had **tuberculosis** on your farm, did he/she drink raw milk/milk products?
  - o 1. Yes |__| 2. No |__|
- ❖ If anyone has had **tuberculosis** on your farm, did he/she eat raw meat?
  - o 1. Yes |__| 2. No |__|
